# Gun Carrying Among Youths, by Demographic Characteristics, Associated Violence Experiences, and Risk Behaviors — United States, 2017–2019

**DOI:** 10.15585/mmwr.mm7130a1

**Published:** 2022-07-29

**Authors:** Thomas R. Simon, Heather B. Clayton, Linda L. Dahlberg, Corinne David-Ferdon, Greta Kilmer, Colleen Barbero

**Affiliations:** ^1^Division of Violence Prevention, National Center for Injury Prevention and Control, CDC; ^2^Public Health Informatics Office, Center for Surveillance, Epidemiology and Laboratory Services, CDC; ^3^Division of Adolescent and School Health, National Center for HIV, Viral Hepatitis, STD, & TB Prevention, CDC.

Suicide and homicide are the second and third leading causes of death, respectively, among youths aged 14–17 years (*1*); nearly one half (46%) of youth suicides and most (93%) youth homicides result from firearm injuries (*1*). Understanding youth gun carrying and associated outcomes can guide prevention initiatives (*2*). This study used the updated measure of gun carrying in the 2017 and 2019 administrations of CDC’s Youth Risk Behavior Survey* (YRBS) to describe the national prevalence of gun carrying for reasons other than hunting or sport among high school students aged <18 years and to examine the associations between gun carrying and experiencing violence, suicidal ideation or attempts, or substance use. Gun carrying during the previous 12 months was reported by one in 15 males and one in 50 females. Gun carrying was significantly more likely among youths with violence-related experiences (adjusted prevalence ratio [aPR] range = 1.5–10.1), suicidal ideation or attempts (aPR range = 1.8–3.5), or substance use (aPR range = 4.2–5.6). These results underscore the importance of comprehensive approaches to preventing youth violence and suicide, including strategies that focus on preventing youth substance use and gun carrying (*3*).

CDC’s YRBS uses an independent three-stage cluster sample design to achieve a nationally representative sample of students in grades 9–12 who attend public or private schools in the 50 states and the District of Columbia (*4*). The overall response rates for 2017 and 2019 were 60% (14,765) and 60.3% (13,677), respectively. After the removal of responses missing age (153; 0.5%), those indicating legal age to purchase a firearm (i.e., age ≥18 years) (3,412; 12%), and those missing sex (138; 0.5%) or gun carrying information (2,927; 10.3%), the final analytic sample included 21,812 students. Information on YRBS weighting, sampling, and psychometric properties has previously been reported (*4**,**5*). YRBS was reviewed and approved by CDC and ICF institutional review boards.^†^

The YRBS gun carrying question was modified in 2017 to exclude carrying for recreational use and to expand the time frame from 30 days to 12 months to permit inclusion of infrequent carrying. Gun carrying was assessed by the question, “During the past 12 months, on how many days did you carry a gun? (Do not count the days when you carried a gun only for hunting or for a sport, such as target shooting).” The question reflects overall gun carrying and is not specific to a particular context such as a school or neighborhood. Gun carrying on school property is not assessed in the national YRBS. Both years of data (2017 and 2019) with the same new wording were used to maximize the sample size for analyses with relatively rare experiences and risk behaviors. The prevalence of gun carrying was comparable across years. Responses were coded as zero days versus ≥1 days (1 to ≥6 days), and prevalence differences were examined by sex, race and ethnicity, age, and sexual identity (i.e., heterosexual, gay/lesbian/bisexual, or not sure). Chi-square and t-tests were used to assess demographic differences, with p-values <0.05 considered statistically significant. Associations between gun carrying and 17 independent variables reflecting experiences with violence, suicidal ideation or attempts, or substance use (Supplementary Table 1, https://stacks.cdc.gov/view/cdc/119459) were assessed in separate sex-stratified adjusted logistic regression models, which generated aPRs and corresponding 95% CIs for each independent variable. All regression models included age, race and ethnicity, and sexual identity. SUDAAN statistical software (version 11.0.1; RTI International) accounted for the complex sample design and weighting of the survey. Frequency of gun carrying was examined among 766 male and 209 female students who carried a gun on ≥1 day in the 12 months preceding the survey. Similar models were used to test differences between those who carried a gun on ≥6 days compared with those who carried a gun on 1–5 days.

Gun carrying was significantly more prevalent among males (6.8%) than among females (1.9%) (Table 1). Among males, gun carrying was most common among non-Hispanic Black (Black) students (10.6%), followed by Hispanic (7.2%) and non-Hispanic White (White) (6.1%) students. Among females, gun carrying was more common among Hispanic (3.5%) than among Black (2.0%) and White students (1.1%).

**TABLE 1:** Prevalence of gun carrying among high school students aged <18 years (N = 21,812), by demographic characteristics — National Youth Risk Behavior Survey, United States, 2017 and 2019

Characteristic	Males (n = 10,521)			Females (n = 11,291)		
	% (95% CI)		Chi-square p-value	% (95% CI)		Chi-square p-value
	0 days	≥1 day		0 days	≥1 day	
**Total**	**93.2 (92.4–93.9)**	**6.8 (6.1–7.6)**	**—**	**98.1 (97.6–98.5)**	**1.9 (1.5–2.4)**	**—**
Race and ethnicity*						
Black^†^	89.4 (86.7–91.6)	10.6 (8.4–13.3)^§^	0.001	98.0 (96.8–98.8)	2.0 (1.2–3.2)	0.003
White^†^	93.9 (92.7–94.9)	6.1 (5.1–7.3)		98.9 (98.4–99.2)	1.1 (0.8–1.6)	
Hispanic	92.8 (91.5–93.9)	7.2 (6.1–8.5)^¶^		96.5 (94.9–97.6)	3.5 (2.4–5.1)^¶,^**	
**Age group, yrs**						
≤15	93.7 (92.7–94.5)	6.3 (5.5–7.3)	0.170	97.6 (96.8–98.3)	2.4 (1.7–3.2)	0.028
16–17	92.8 (91.8–93.8)	7.2 (6.2–8.2)		98.5 (97.9–98.8)	1.5 (1.2–2.1)^††^	
**Sexual identity**						
Heterosexual	93.6 (92.7–94.3)	6.4 (5.7–7.3)	0.290	98.4 (97.9–98.8)	1.6 (1.2–2.1)	0.098
Gay, lesbian, or bisexual	94.1 (90.7–96.3)	5.9 (3.7–9.3)		97.6 (96.4–98.4)	2.4 (1.6–3.6)	
Not sure	89.7 (83.8–93.6)	10.3 (6.4–16.2)		95.6 (91.2–97.8)	4.4 (2.2–8.8)	

* Other races and ethnicities are not presented because of limited interpretability of these heterogenous groups.

^†^ Non-Hispanic.

^§^ Significant difference between White and Black students based on t-test analysis (p<0.05).

^¶^ Significant difference between Black and Hispanic students based on t-test analysis (p<0.05).

** Significant difference between White and Hispanic students based on t-test analysis (p<0.05).

^††^ Significant difference between students aged ≤15 years and those aged 16–17 years, based on t-test analysis (p<0.05).

Gun carrying was significantly more prevalent among those students who had experienced violence, suicidal ideation or attempts, or substance use than it was among those who had not (Table 2). For example, gun carrying among males and females was more prevalent among those who had been threatened or injured with a weapon on school property (25.9% and 11.2%, respectively) than it was among those who had not (5.2% and 1.3%, respectively). The aPRs for all 10 violence-related experiences, including fighting, bullying, dating violence, missing school because of safety concerns, and sexual violence, were significant (aPR ranges = 1.6–6.3 and 1.5–10.1 among males and females, respectively). Gun carrying was significantly more prevalent among students who reported seriously considering attempting suicide (aPR for males = 1.9; aPR for females = 1.8) or attempting suicide (aPR for males = 3.1; aPR for females = 3.5) than it was among those who had not. Each substance use measure was associated with higher prevalence of gun carrying (aPR ranges = 4.2–5.2 and 4.3–5.6 among males and females, respectively). Students who had been offered or sold drugs on school property were also more likely to carry a gun (aPR for males = 2.8; aPR for females = 4.0).

**TABLE 2:** Prevalence of gun carrying, by violence, suicide, and substance use–related behaviors and experiences among high school students aged <18 years, by sex — National Youth Risk Behavior Survey, United States, 2017 and 2019

Risk behaviors and experiences	Males			Females		
	Carried a gun, % (95% CI)		aPR* (95% CI)	Carried a gun, % (95% CI)		aPR* (95% CI)
	Did not experience the risk behavior	Experienced the risk behavior		Did not experience the risk behavior	Experienced the risk behavior	
In a physical fight^†^	2.9 (2.3–3.6)	15.4 (13.3–17.8)	5.6 (4.3–7.2)	0.7 (0.5–1.0)	7.0 (5.4–8.9)	10.1 (6.2–16.3)
In a physical fight on school property^†^	4.8 (4.1–5.6)	21.0 (18.2–24.0)	4.3 (3.5–5.4)	1.3 (1.0–1.7)	11.7 (8.6–15.7)	8.0 (5.4–11.8)
Threatened or injured with a weapon on school property^†^	5.2 (4.6–6.0)	25.9 (21.7–30.6)	5.0 (4.0–6.1)	1.3 (1.0–1.8)	11.2 (7.5–16.4)	6.9 (4.3–11.1)
Was electronically bullied^†^	6.2 (5.4–7.1)	11.3 (9.5–13.4)	2.0 (1.6–2.6)	1.5 (1.1–2.0)	3.3 (2.5–4.4)	2.3 (1.6–3.2)
Was bullied on school property^†^	6.3 (5.5–7.2)	9.1 (7.8–10.7)	1.6 (1.3–2.0)	1.6 (1.3–2.1)	2.6 (1.9–3.5)	1.5 (1.1–2.1)
Missed school because felt unsafe^§^	5.8 (5.0–6.7)	20.8 (16.7–25.7)	3.6 (2.7–4.8)	1.4 (1.1–1.8)	7.4 (5.1–10.7)	4.8 (3.0–7.6)
Carried a weapon^¶^ on school property^§^	5.3 (4.6–6.2)	34.3 (28.5–40.5)	6.3 (5.0–8.1)	1.5 (1.2–2.0)	21.1 (13.2–32.2)	10.1 (6.0–17.0)
Experienced sexual violence by anyone^†^	5.7 (4.9–6.6)	24.0 (19.1–29.8)	4.1 (3.2–5.4)	1.1 (0.9–1.5)	5.7 (4.3–7.4)	5.0 (3.6–6.9)
Experienced sexual dating violence^†,^**	7.3 (6.4–8.5)	33.0 (24.9–42.3)	4.7 (3.5–6.3)	1.8 (1.3–2.4)	5.9 (4.2–8.3)	2.9 (2.0–4.4)
Experienced physical dating violence^†,††^	7.5 (6.5–8.6)	22.9 (18.2–28.5)	3.3 (2.5–4.3)	1.8 (1.4–2.4)	7.5 (5.3–10.6)	3.0 (2.0–4.5)
Seriously considered attempting suicide^†^	6.1 (5.3–7.0)	11.7 (9.6–14.1)	1.9 (1.5–2.5)	1.5 (1.1–2.0)	3.3 (2.5–4.3)	1.8 (1.3–2.6)
Attempted suicide^†^	5.8 (5.0–6.7)	19.4 (14.4–25.6)	3.1 (2.2–4.5)	1.3 (1.0–1.8)	5.7 (4.0–8.0)	3.5 (2.3–5.3)
Current binge drinking^§§^	4.0 (3.5–4.6)	18.7 (15.6–22.2)	5.2 (4.1–6.7)	1.0 (0.7–1.5)	5.3 (3.9–7.2)	5.6 (3.4–9.0)
Current marijuana use^¶¶^	4.0 (3.4–4.8)	16.9 (14.7–19.5)	4.2 (3.4–5.2)	1.0 (0.8–1.3)	4.8 (3.4–6.7)	4.8 (3.2–7.1)
Lifetime prescription drug misuse***	4.5 (3.9–5.1)	22.6 (19.4–26.1)	5.2 (4.3–6.3)	1.2 (0.9–1.6)	5.8 (4.6–7.2)	4.3 (3.1–6.1)
Lifetime illicit drug use^†††^	4.6 (3.9–5.5)	19.9 (17.1–23.0)	4.4 (3.5–5.5)	1.1 (0.8–1.4)	6.5 (4.9–8.6)	5.6 (3.9–8.0)
Offered or sold drugs on school property^†^	4.6 (4.0–5.3)	13.6 (11.6–15.9)	2.8 (2.3–3.4)	1.0 (0.7–1.5)	5.0 (3.9–6.3)	4.0 (2.7–6.1)

**Abbreviation:** aPR = adjusted prevalence ratio.

* Models adjusted for age, race and ethnicity, and sexual identity.

^†^ During the 12 months before the survey.

^§^ On ≥1 day during the 30 days before the survey.

^¶^ Such as a gun, knife, or club.

** Among students who dated or went out with someone during the 12 months before the survey and answered the sexual dating violence question (6,573 males; 7,094 females).

^††^ Among students who dated or went out with someone during the 12 months before the survey and answered the physical dating violence question (7,385 males; 8,194 females).

^§§^ Had four or more drinks of alcohol in a row (if they were female) or five or more drinks of alcohol in a row (if they were male) within a couple of hours on ≥1 day during the 30 days before the survey.

^¶¶^ One or more times during the 30 days before the survey.

*** One or more times during the respondent’s lifetime.

^†††^ Lifetime use of at least one of the following: heroin, cocaine, methamphetamines, synthetic marijuana, ecstasy, hallucinogenic drugs, or inhalants.

Most students who carried a gun reported carrying on 1–3 days (males = 46.8%; females = 69.8%) or ≥6 days (males = 42.0%; females = 21.6%) during the past 12 months (Figure). Overall, those who carried a gun on ≥6 days were more likely to report three of the violence-related experiences, suicidal ideation or attempts, and all four substance use measures than were those who carried a gun less often (Supplementary Table 2, https://stacks.cdc.gov/view/cdc/119473).

**FIGURE:**
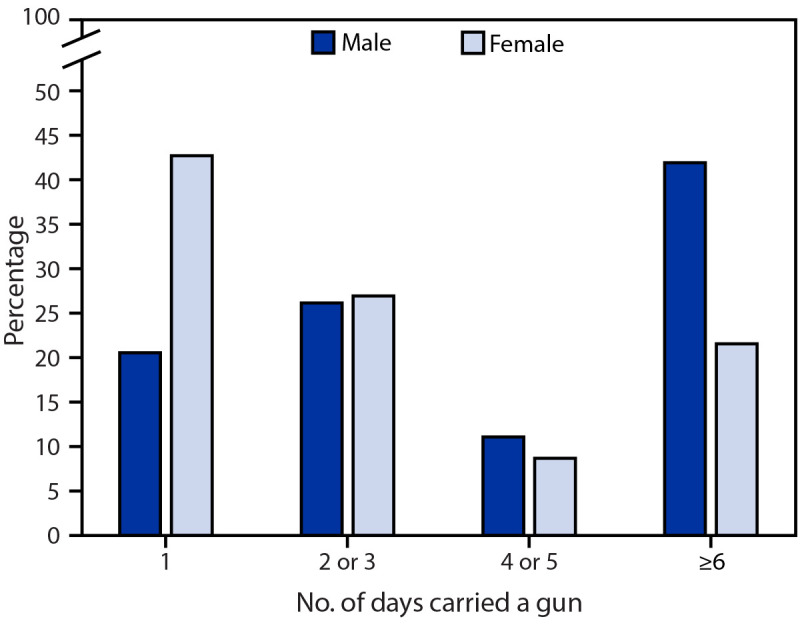
Frequency of gun carrying among high school students aged <18 years (males, n = 766; females, n = 209) who carried a gun ≥1 day during the past 12 months, by sex — National Youth Risk Behavior Survey, United States, 2017 and 2019

## Discussion

The revised YRBS question helps distinguish potentially risky forms of gun carrying from recreational use, and the expanded time frame allows infrequent gun carrying by youths to be included. Whereas one in 15 males and one in 50 females carried a gun at least once in the 12 months before the survey, the prevalence of gun carrying was much higher among some subgroups of youths, particularly those who missed school because of safety concerns and those who had experienced violence. For example, among those who were threatened or injured with a weapon on school property, more than one in four males and one in nine females carried a gun. Youths who carried a gun more frequently were more likely to have engaged in substance use and to have experienced violence. Youths who carry guns often report self-protection as the reason; however, youth gun carrying is associated with risk for serious injury or death (*2**,**6*). The higher prevalence of gun carrying among those who have experienced suicidal ideation or attempts or other forms of violence highlights the potential for lethal consequences if firearms are used against oneself or others. The association between youth gun carrying and substance use further suggests an increased risk for impaired, impulsive, situational, or escalating actions (*7*).

When variations in gun carrying across racial and ethnic groups and in relation to youth behaviors and experiences are reviewed, consideration of the larger context is important. Social and structural conditions (e.g., concentrated poverty, high crime rates, and economic or residential instability) are associated with youth violence and contribute to inequities in violence among racial and ethnic minority populations (*3*). Further, youths who have experienced violence, discrimination, or racism might feel an increased need for protection, might be unwilling or unable to rely on law enforcement, and might carry a gun for self-protection (*2**,**6*).

The findings in this report are subject to at least four limitations. First, YRBS data are cross-sectional and cannot be used to determine the temporal order of associations. Second, all examined behaviors, including gun carrying, were self-reported and therefore might be misreported. Third, the category of students unsure of their sexual identity might include students who are not yet certain of their sexual identity and students who did not understand the question (*4*). Finally, YRBS does not collect contextual factors that might elucidate the gun carrying behaviors of youth (e.g., how acquired, where carried, substance use while carrying, and carrying a gun for someone else).

These findings suggest that a substantial proportion of high school students, particularly those who have experienced violence, suicidal ideation or attempts, or who engage in substance use, carry guns outside the context of hunting or sport. Some studies have found that counseling and education with provision of safety devices can promote safer firearm storage behaviors in the home and that child access prevention laws are associated with reductions in risk for firearm suicide, unintentional firearm injuries, and gun carrying among children and youths *(**8**–**10**)*. However, additional research is necessary to identify strategies to prevent youth gun carrying and support effective implementation of such strategies, especially among those youths at highest risk for experiencing violence. Taken together, the results underscore the importance of comprehensive approaches to preventing multiple forms of violence affecting youths and associated behaviors such as substance use and gun carrying. To help states and communities take advantage of the best available evidence to prevent violence, CDC has released a series of technical packages that describe the evidence for programs, policies, and practices to reduce multiple forms of violence, including youth violence, sexual or dating violence, and suicide, through strategies such as connecting youths to caring adults and activities, strengthening economic supports, improving access and delivery of care, creating protective environments, and teaching coping and problem-solving skills (*3*).

**Summary**

**What is already known about this topic?**

Among youths aged 14–17 years, suicide and homicide are the second and third leading causes of death, respectively. Most youth homicides result from firearm injuries; firearms are the most common method of youth suicide.

**What is added by this report?**

Using a new measure that excludes recreational gun carrying, one in 15 male and one in 50 female high school students reported carrying a gun for nonrecreational purposes at least once during the preceding 12 months. Gun carrying was more prevalent among those who experienced violence, suicidal ideation or attempts, or substance use.

**What are the implications for public health practice?**

Comprehensive strategies using the best available evidence including addressing youth substance use and gun carrying can prevent youth violence and suicide.

## Footnotes

*
https://www.cdc.gov/healthyyouth/data/yrbs/results.htm

† 45 C.F.R. part 46; 21 C.F.R. part 56.
